# Metagenomic analysis and the functional profiles of traditional fermented pork fat ‘*sa*-*um*’ of Northeast India

**DOI:** 10.1186/s13568-018-0695-z

**Published:** 2018-10-08

**Authors:** Surajit De Mandal, Sambanduram Samarjit Singh, Rajendra Bose Muthukumaran, Kawl Thanzami, Vinod Kumar, Nachimuthu Senthil Kumar

**Affiliations:** 1 Department of Biotechnology, Mizoram University, Aizawl, Mizoram 796004 India; 2 Department of Chemistry, Mizoram University, Aizawl, Mizoram 796004 India; 3 Dept of Pharmacy, Regional Institute of Paramedical and Nursing Sciences, Aizawl, Mizoram 796017 India; 4 Biotech Consortium India Ltd, Anuvrat Bhawan, Deen Dayal Upadhyaya Marg, New Delhi, 110002 India

**Keywords:** Pork fat, *Sa*-*um*, Nutrient content, Bacterial community, Food safety

## Abstract

Fermented pork fat (*sa*-*um*) is traditionally and extensively consumed in Northeast Indian region for several decades. However, no scientific reports are available regarding its nutritional value as well as its potential health risks. The objective of this work was essentially the characterization of *sa*-*um* using a polyphasic approach, viz., physicochemical, electrospray ionization-mass spectrometry (ESI^**+**^-MS) and metagenomic analysis in order to gain an understanding of the nutrient contents and microbial population diversity. On a dry weight basis, about 91% fat, 2% carbohydrate and 0.70% protein were present. ESI^**+**^-MS analysis of *sa*-*um* revealed the presence of various polar and neutral lipids corresponding to monoacylglyceride, diacylglyceride and triacylglyceride species. The dominant bacterial phyla were *Firmicutes*, *Proteobacteria* and *Bacteroidetes*. A total of 72 bacterial genera were identified, largely abundant with *Clostridium* species including *C. butyricum*, *C. citroniae*, *C. methylpentosum*, *C. perfringens*, *C. saccharogumia* and *C. tetani*. The imputed functional profiles of bacterial communities were predominantly involved in energy, carbohydrate and amino acid metabolisms. Furthermore, this study deduces the presence of pro-inflammatory molecules as well as antibiotic resistance genes associated with the bacterial families such as *Bacillaceae*, *Bacteroidaceae*, *Clostridiaceae*, *Corynebacteriaceae* and *Enterobacteriaceae* which might be a major health concern for the *sa*-*um* consuming population.

**Electronic supplementary material:**

The online version of this article (10.1186/s13568-018-0695-z) contains supplementary material, which is available to authorized users.

## Introduction

Fermentation is regarded as an ancient and economical method for value addition and preservation of foodstuff in Northeast India (Tamang 1998). The indigenous foods are used from ancient times and directly related with the tradition and culture (Sekar and Mariappan 2007). However, the preparation of indigenous or traditional fermented foods only remains as a household art today (Beuchat 2008). The nutritional value of the food is concomitantly augmented with the increase in vitamin content and protein solubility during fermentation (Sohliya et al. 2009). Fermentation also serves as a potential source of bioactive compounds which may provide antimicrobial, cholesterol-lowering ability as well as antithrombotic and antioxidative activities (Hartmann and Meisel 2007). Diverse ethnic communities of Northeast India prepare various kinds of fermented food products and use them as a basic component of their diet (Tamang et al. 2009). Fermented pork fat is being consumed by people from different parts of the world as a source of daily food (Aquilanti et al. 2007). *Sa*-*um*, an indigenous animal fat product is semi-dry, gummy, ‘ripened’ lard made with caul fat adipose tissue and it has no appreciable organoleptic qualities although it exhibits distinct astringency. It presents negative health attributes due to high saturated fat/cholesterol content as it is derived from pork fat (Hooper et al. 2001). *Sa*-*um* preparation takes place on a cottage-industrial scale in households which does not have firmly established procedures and as a result the production process fluctuates on a seasonal basis. *Sa*-*um*, has peculiar sensorial attributes (smell and taste) due to ripening process besides the enzymatic lipolytic activities of the microbial populations present in it. Pork and beef products are often associated with different microbial populations (Borch et al. 1996; Nieminen et al. 2011; Pennacchia et al. 2011). However, no detailed study has been carried out on the chemical or microbiological characteristics of *sa*-*um*.

Exploration of bacterial community using conventional methods provides limited information than the actual diversity. In recent years, next generation sequencing (NGS) technology has considerably enhanced our ability to assess and understand the microbial communities, and it provides a better understanding of the complex interactions among the diverse bacterial species in a specific community. NGS has been employed to investigate the microbial communities of various foods (Solieri et al. 2013; Mayo et al. 2014).

Mass spectrometry methods are rapid, robust, and reliable tool for the lipid profiling in plant and animal derived fat products (Goodacre et al. 2002; Kurata et al. 2005). In this study, we report the taxonomic composition of microbial community and their putative metabolic functions in *sa*-*um* and also compared with the other published fermented pork metagenomes (Połka et al. 2014). This is the first attempt to evaluate the nutrient content as well as bacterial diversity of *sa*-*um*, and to ascertain the nutritional value and safety attributes with respect to human health.

## Materials and methods

### Preparation method of *sa*-*um* and sample collection

**Fig. 1:**
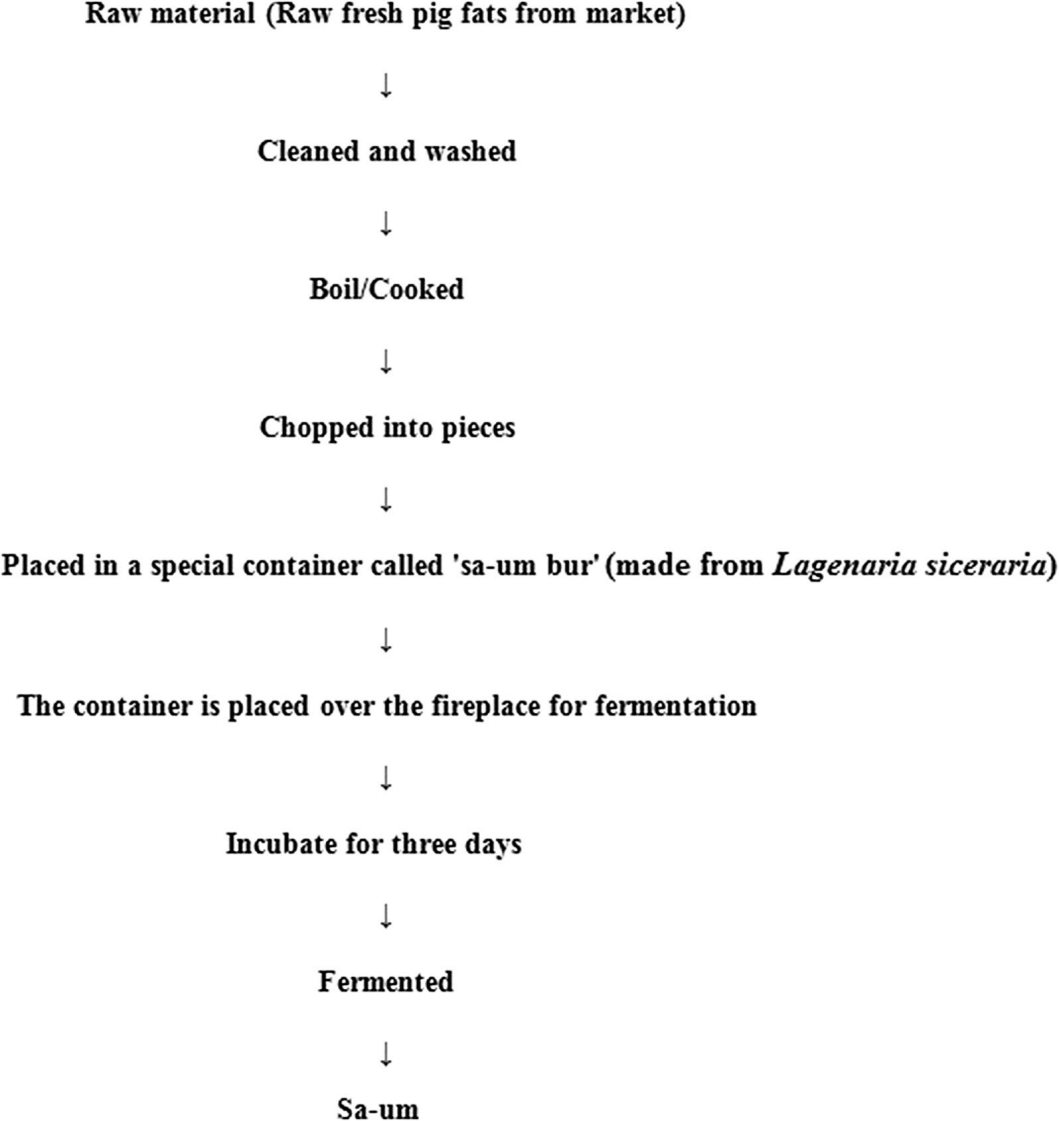
Traditional method of *sa*-*um* preparation

**Fig. 2:**
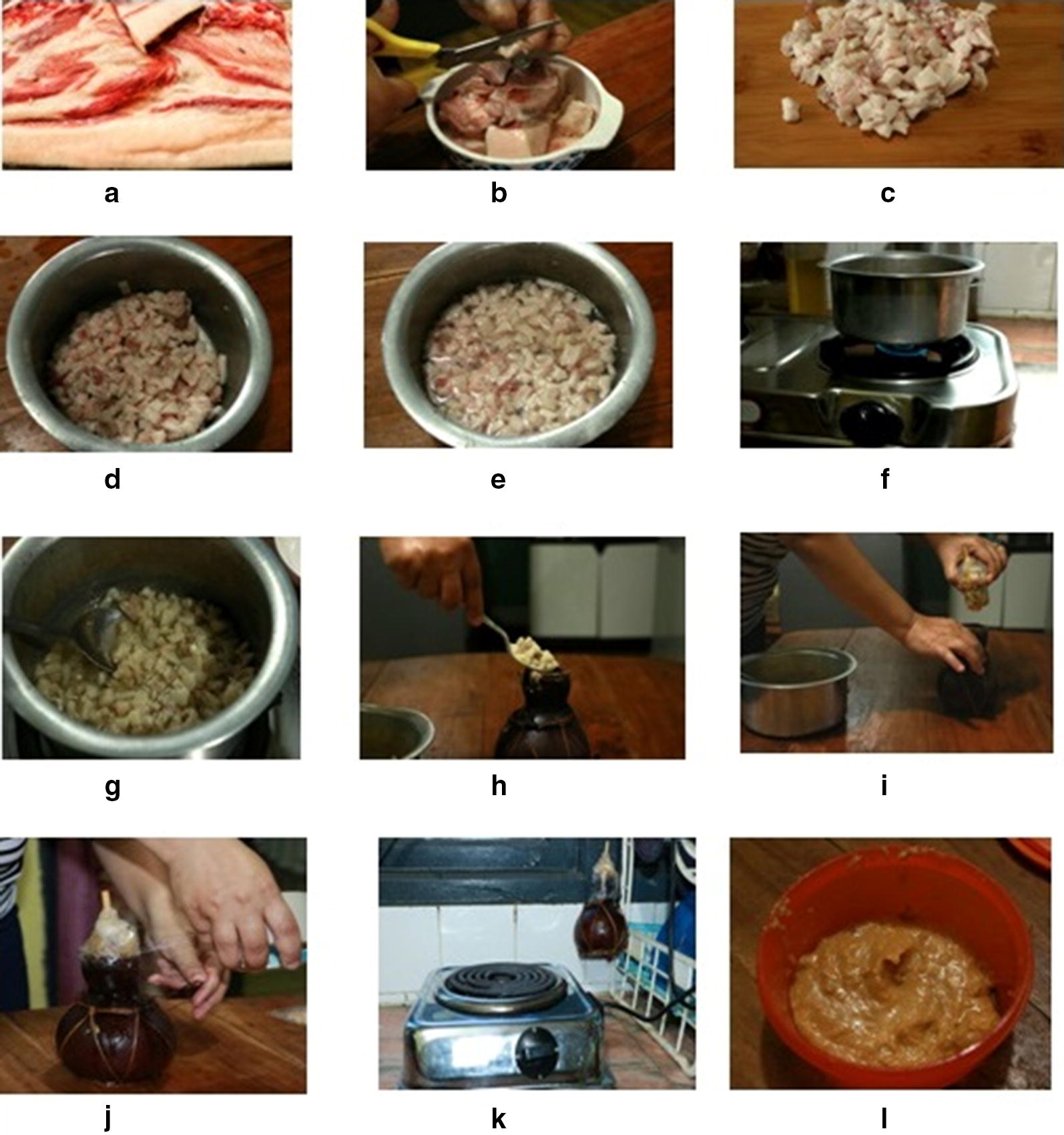
Different steps involved in *sa*-*um* preparation. **a** Pork lard, **b**–**d** mincing of lard, **e**–**g** added water and cooked till water evaporates out, **h**–**j** cooked lard transferred to cleansed and dried ‘Um’, **k** incubated at fireplace or sun, **l** finished product *sa*-*um*

Caul fat adipose tissue is normally obtained from butcher’s yard, within 6 h of slaughtering the animal, boiled with minimum amount of water for about 15 min (rendering) and allowed to cool. When caul fat is not available, sub-cutaneous fat adipose tissue is also employed for the production process. Boiled caul fat adipose tissue is transferred to a dry bottle gourd container for ‘ripening’ of the adipose tissue. The container is kept for 3–5 days under the sun for ripening during dry winter season, while it may be kept near cooking stove (~ 10–15 cm from cooking stove) for 2–3 days during rainy monsoon season. After the ripening and dehydration process, consequently the caul fat loses most of its natural and added water content and it becomes semi-dry with gummy, soft, and spongy texture. Traditional method of production of *sa*-*um* is given in Figs. 1, 2. Six *sa*-*um* samples (200 g) were collected from different parts of Mizoram, Northeast India, and mixed thoroughly in a sterile 500 ml polypropylene container. Collected samples were brought immediately to the laboratory in cold box, stored at 4 °C and subsequently used for analysis within 1–2 days of collection.

### Biochemical analysis

Moisture, ash, crude fiber, protein, fat, carbohydrates, calorific value, iron, zinc, sodium, calcium, magnesium, potassium content of *sa*-*um* were determined according to AOAC methods (AOAC 1995). The pH of samples was determined using a pH meter (Eutech, India).

### Mass spectrometry analysis

2.5 g of *sa*-*um* samples were kept in dichloromethane: methanol or acetonitrile: methanol as solvent systems for overnight followed by sonication for 10 min. The samples were vortexed for another 5 min and centrifuged. The organic solution layer was subjected to mass spectrometry analysis. ESI-tandem mass spectrometry measurements were carried out with a Waters QTOF-Micromass spectrometer with nitrogen as carrier gas (flow rate 100 l/hr) and the sample flow rate was 0.2 ml/min with the desolvation temperature of 150 °C. Mass Spectra were recorded by electrospray ionization and the source voltage was maintained at 2.3 kV in the positive ion mode.

HP-TLC analysis was performed to analyze the lipid component present in *sa*-*um*.

### DNA isolation, PCR and sequencing

Isolation of the metagenomic DNA from *sa*-*um* samples were carried out using the Fast DNA spin kit (MP Biomedical, USA) and quantified by microplate reader (Spectra Max 2E, Molecular Devices, USA). The V4 hyper variable region of the 16S rRNA gene was amplified by 10 pmol/μl of each forward 515F (5′-3′) and reverse 806R (5′-3′) primers. The amplification mix contained 40 mM dNTPs (NEB, USA); 5× Phusion HF buffers (NEB, USA); 2 U/μl F-540 Special Phusion HS DNA Polymerase (NEB, USA); and 5 ng DNA and Milli-Q water to make up 30 μl total volume. PCR conditions consisted of initial denaturation at 98 °C for 30 s followed by 30 cycles of 98 °C for 10 s, 72 °C for 30 s, and a final extension at 72 °C for 5 s. Paired end Illumina MiSeq sequencing (2 × 250 bp) was performed at SciGenom Lab, Cochin, India (De Mandal et al. 2016, 2017).

### Sequencing analyses

Raw fastq sequences were processed and analyzed using the QIIME software package v.1.8.0. (Caporaso et al. 2009, 2010). Sequences with a quality score < 25 and read length < 200 bp were filtered and chimeric sequences were remove using USEARCH (Edgar et al. 2011). Preprocessed V4 sequences were clustered into operational taxonomic units (OTU’s) using the Uclust program (similarity cutoff = 0.97) (Edgar 2010). Representative sequence for each OTU was classified using Greengenes database (De Mandal et al. 2016, 2017; DeSantis et al. 2006).

### Imputed functional analysis

The imputed functional profile of the bacterial community was performed using PICRUSt (Langille et al. 2013). In brief closed-reference, OTU was generated at a 97% similarity level and normalized using “normalize_by_copy_number.py” script. Functional profiles were predicted against KEGG database. Contributions of various taxa to different KOs were computed with the script metagenome_contributions.py (Langille et al. 2013).

### Comparison of *sa*-*um* with Italian salami

Metagenome data was retrieved from SRA (Sequence Read Archives) NCBI in fastq format to compare present studies with the previously published fermented pork (Italian salami) (Połka et al. 2014). Only the samples with no starter culture (SRR5298557, SRR5298565, SRR5298588, SRR5298586) were used in our study and the good quality sequences were compiled into a single fasta file and processed using QIIME pipeline. The compositional similarity between the fermented pork metagenome was compared using Bray–Curtis measure for estimation of beta diversity (Bray and Curtis 1957).

## Results

### Biochemical and mass spectrometry analysis of *sa*-*um*

**Fig. 3:**
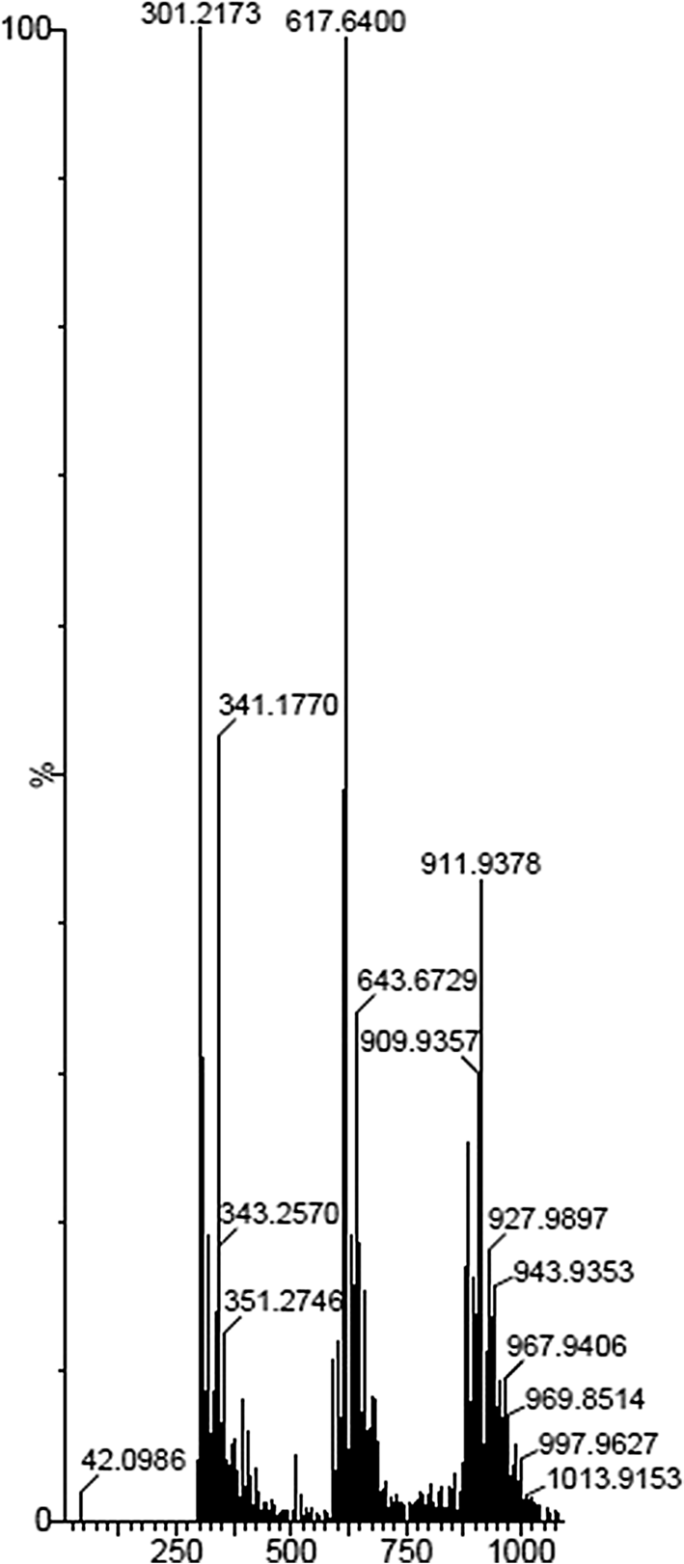
Mass spectral trace of *sa*-*um* by ESI^+^-MS method. m/z: 300-351-monoacyl glycerol, with higher intensity around m/z: 300–304 region due to arachidonic acid, typical fatty acid of non-ruminant species; m/z: 550–650-diacyl glycerol species; m/z: 750–850-triacyl glycerol species; with a modest levels of triacyl phospholipid species in the m/z: 900 region, due to smaller quantity of muscle adipose tissues in *sa*-*um*

As a major constituent, the fat content accounted for 91% followed by moisture content (6.21%) besides trace amounts of carbohydrate, crude fiber and ash. The high fat content of *sa*-*um* is reflected in its calorific value (830 kcal/100 g) and the pH was slightly acidic (6.60). Physicochemical compositions of the fermented pork fat *sa*-*um* are summarized in Table 1. Preliminary ESI^**+**^-MS analysis of *sa*-*um* has indicated the presence of various polar and neutral lipid molecular species in the range from 295 to 920 Dalton and these lipid components are essentially clustered in the mass range of 300, 600, 900 that corresponds to monoacylglyceride, diacyl-glyceride, and triacylglyceride molecular species with a noticeable presence of the myristic acid (C14:0) in acylglycerides besides sterols (Fig. 3). The observed glycerides consist of acyl sidechains corresponding to fourteen carbons or longer, while the neutral lipid molecular components comprise unsaturated fatty acid glycerides (mono and poly) as well as saturated fatty acid containing acylglycerides. HP-TLC analysis showed the presence of lipid components with a wide range of polarity that commensurate with the mass spectrometric analysis of *sa*-*um* (Additional file 1: Figure S1).

**Table 1:** Biochemical analysis of *sa-um*

	Value
Major nutrients (% by wt.)	
Moisture	6.21
Total ash	0.1
Fat	91
Crude fiber	BDL of 0.1
Protein	0.7
Carbohydrates	2
Calorific value (Kcals/100 g)	830
Minerals (mg/100 g)	
Iron	0.2
Zinc	0.07
Sodium	4.73
Calcium	2.61
Magnesium	0.45
Potassium	3.96

*BDL* below detection limit

### Phylogenetic analysis of the bacterial community

**Fig. 4:**
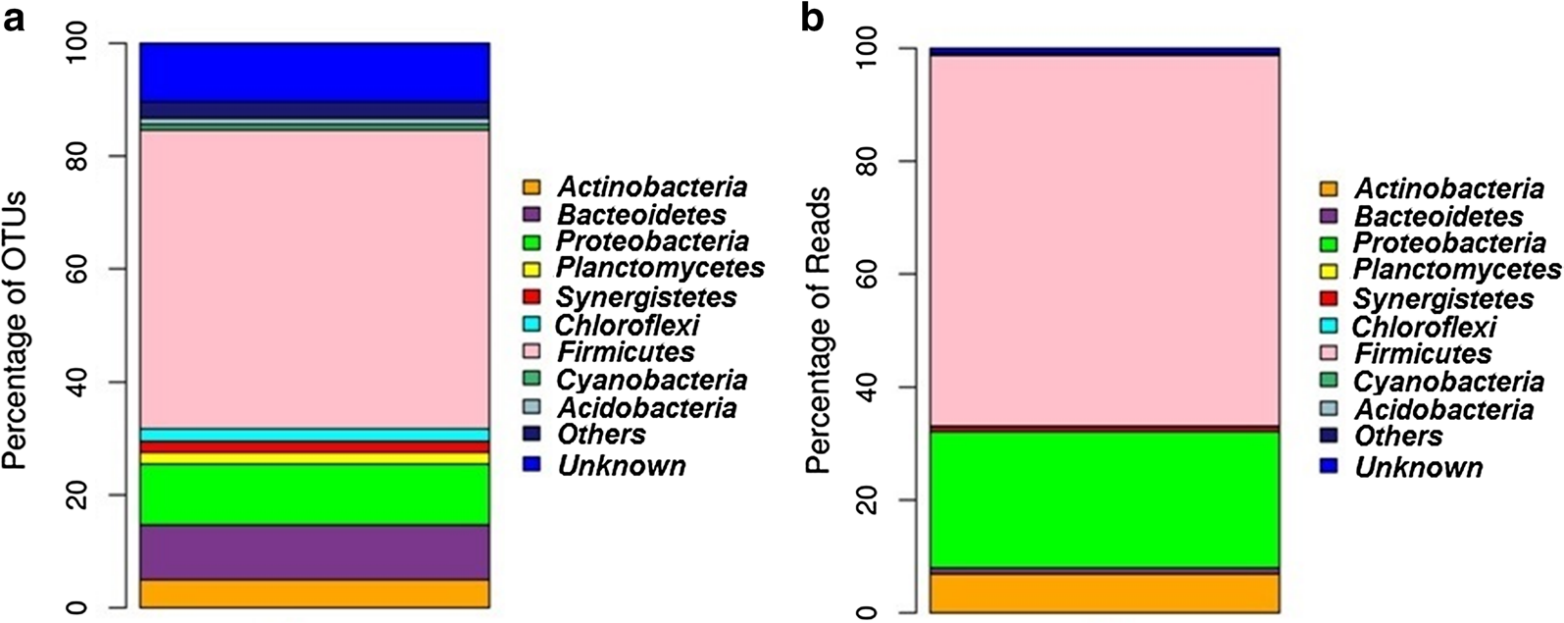
Taxonomy classification of OTU’s at phylum level (**a**) and reads at phylum level (**b**) from *sa*-*u*m. Only top 10 enriched phyla categories are shown in the figure

A total of 263,741 paired-end raw reads were obtained from the *sa*-*um* metagenome. After pre-processing, 60,117 reads were obtained and clustered into 1348 OTU’s with 97% sequence similarity. Further analyses detected 627 singletons and were subsequently removed from the OTU table. Finally, 722 OTU’s were taken for downstream analysis (Table 2). Dominant phyla were *Firmicutes* (52.91%), *Proteobacteria* (10.8%), *Bacteroidetes* (9.6%), *Actinobacteria* (4.98%), *Chloroflexi* (2.21%), *Planctomycetes* (2.07%), *Synergistetes* (1.193%) and *Acidobacteria* (1.1%). Other minor identified phyla (< 1%) were *Cyanobacteria*, *Verrucomicrobia*, *AD3*, *Thermi*, *Armatimonadetes*, *Tenericutes*, *Crenarchaeota*, *GAL15*, *Gemmatimonadetes*, *Euryarchaeota*, *Nitrospirae* and *Chlorobi*. However, more than 10% of the total OTU’s remained unidentified. The present study identified 722 OTU’s belonging to three major groups of bacteria: *Firmicutes*, *Proteobacteria and Bacteroidetes* (Fig. 4).

**Table 2:** Raw read summary of *sa-um* using Illumina sequencing

Total reads (paired-end)	263,741
Sequence length (bp)	250
Total data (Mb)	131.87
%GC	52.74
Average base quality (phred score)	35.11
Passed conserved region filter	172,188
Passed spacer	171,871
Passed read quality filter	170,843
Passed mismatch filter	60,117
Consensus reads	60,117
Chimeric sequences	0
Pre-processed reads	60,117
Total OTUs picked	1348
Total singleton OTUs	627
Total OTUs after singleton removal	722

Firmicutes essentially occupied more than half of total OTU’s present in the complete dataset. 82 OTU’s (52.91%) consisted of 39,046 (65.63%) reads assigned under the phylum *Firmicutes*, of which three classes (*Bacilli*, *Clostridia* and *Erysipelotrichia*), six orders (*Clostridiales*, *Bacillales*, *OPB54*, *Lactobacillales*, *Erysipelotrichales* and *Thermoanaerobacterales*), and ten families (*Christensenellaceae*, *Clostridiaceae*, *Enterococcaceae*, *Erysipelotrichaceae*, *Lachnospiraceae*, *Planococcaceae*, *Ruminococcaceae*, *Staphylococcaceae*, *Streptococcaceae* and *Veillonellaceae*) were identified. Top four dominant OTU’s under this genus were *Clostridium tetani* (21.29%), *B. coagulans* (0.47%), *Phascolarctobacterium* sp. (0.32%) and *C. perfringens* (0.31%). Other identified genera includes *Anaerotruncus*, *Blautia*, *Coprobacillus*, *Coprococcus*, *Dialister*, *Dorea*, *Enterococcus*, *Faecalibacterium*, *Lactobacillus*, *Marinococcus*, *Megamonas*, *Megasphaera*, *Oscillospira*, *Phascolarctobacterium*, *Roseburia*, *Ruminococcus*, *Ruminococcus*, SMB53, *Staphylococcus*, *Streptococcus* and *Veillonella*.

Proteobacteria represented the second dominant phyla. A total of 78 (10.8%) OTU’s and 14,371 (24.16%) reads were affiliated with the phylum *Proteobacteria*. Five classes (*Alphaproteobacteria*, *Betaproteobacteria*, *Deltaproteobacteria*, *Epsilonproteobacteria* and *Gammaproteobacteria*) and thirteen orders (*Aeromonadales*, *Alteromonadales*, *Burkholderiales*, *Campylobacterales*, *Desulfobacterales*, *Enterobacteriales*, *Enterobacteriales*, *Oceanospirillales*, *Pseudomonadales*, *Rhizobiales*, *Rhodobacterales*, *Vibrionales* and *Xanthomonadales*) were indentified under this phylum. Dominant OTU’s under this phylum were classified under the genera *Acinetobacter*, *Citrobacter*, *Escherichia*, *Halomonas*, *Proteus* and *Ralstonia.*

The phylum Bacteroidetes consisted of two classes: Bacteroidia and Flavobacteriia. Identified families were *Barnesiellaceae*, *Odoribacteraceae*, *Weeksellaceae*, *Bacteroidaceae*, *Porphyromonadaceae*, *Prevotellaceae*, *Rikenellaceae* and *S24*-*7*. Identified genera under this phylum were *AF12*, *Barnesiella*, *Bacteroides*, *Butyricimonas*, *Parabacteroides*, *Odoribacter*, *Prevotella* and *Wautersiella*. 36 OTU’s comprising 4116 reads belonged to *Actinobacteria*. Identified genera were *Actinomycetospora*, *Adlercreutzia*, *Bifidobacterium*, *Brevibacterium*, *Collinsella*, *Corynebacterium*, *Kocuria*, *Micrococcus* and *Slackia*. The most dominant OTU under this phylum was OTU1271 (6.4%) was classified as *C. variabile*.

**Fig. 5:**
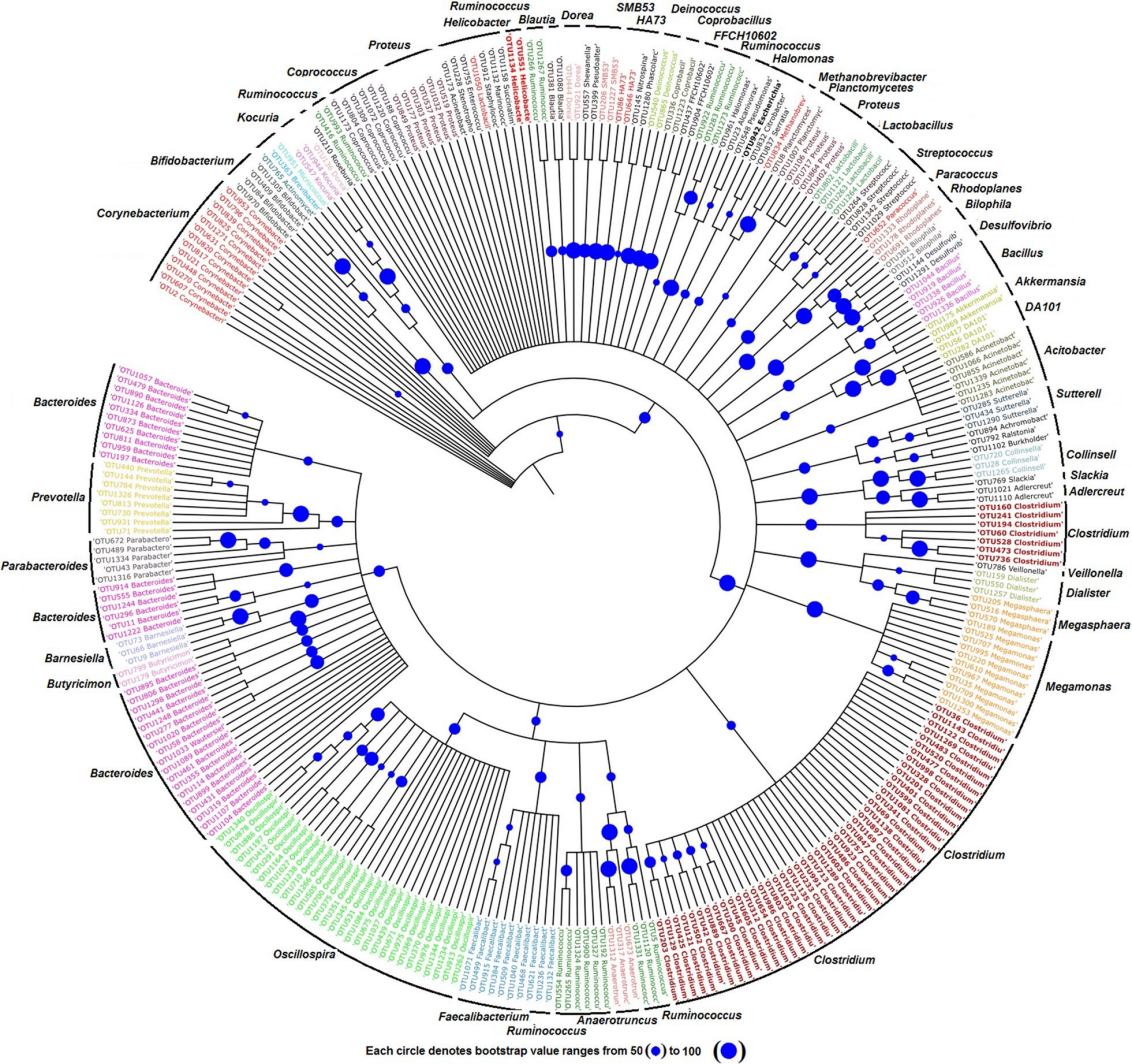
Phylogenetic tree based on the identified genera using neighbour joining method

Two OTU’s were found under the phylum *Chloroflexi* which belonged to *FFCH10602*. Five OTU’s with 34 reads were classified under *Verrucomicrobia*. Identified genera were DA101 and *Akkermansia muciniphila*. Eight OTU’s comprising 34 reads were classified under the phylum *Acidobacteria*. Identified orders include *iii1*-*15*, *RB41*, *Ellin6513*, *RB41*, *Nov*-*24*, *RB41*, *Acidobacteriales* and *Ellin6513*. OTU834 was classified under the genus *Methanobrevibacter* only eukaryobacteria found in this study. Only two OTU’s were classified under *Deinococcus* (phylum *Thermi*). Three OTU’s classified under the phylum AD3. Identified classes were ABS-6 and JG37-AG-4. Two phylotype (OTU 294 and OTU 916) were classified under *Armatimonadetes*. A total of 74 OTU’s identified at the genus level were dominated by *Clostridium* (7.61%), *Bacteroides* (4.57%), *Oscillospira* (4.15%), *Corynebacterium* (1.80%), *Megamonas* (1.52%), *Faecalibacterium* (1.38%), *Proteus* (1.38%), *Ruminococcus* (1.24%), *Prevotella* (1.10%) and unknown genus (58.31%). Phylogenetic tree based on the taxonomically identified genera is shown in Fig. 5.

### Imputed functional analysis

**Fig. 6:**
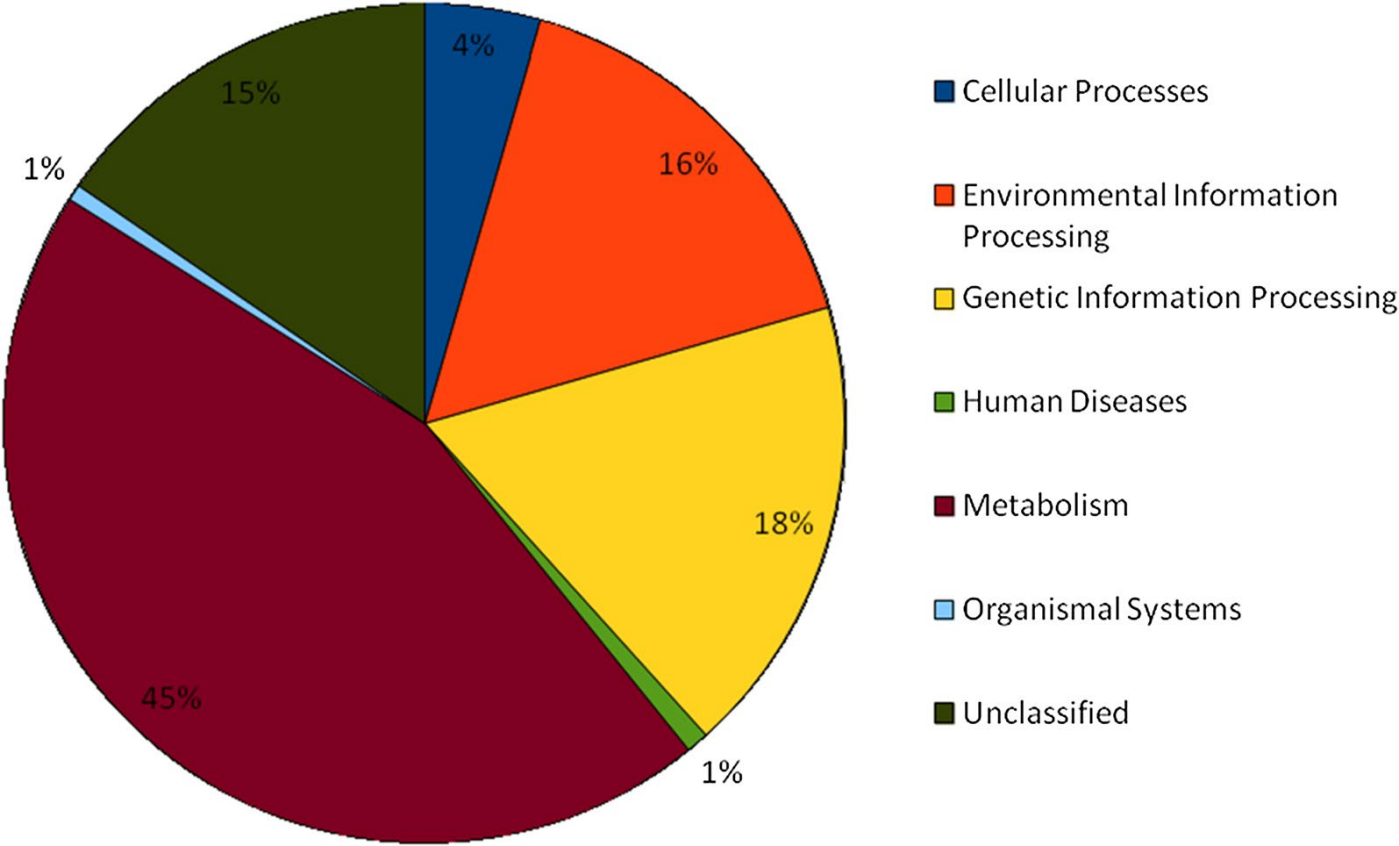
Distribution of KEGG pathway in *sa*-*um* metagenome

Based on the predicted metagenomes, major gene families (45%) belonged to metabolic pathways involved in amino acid, carbohydrate, energy, nucleotide, cofactors and vitamins, lipid and xenobiotics biodegradation metabolism, enzyme families, glycan biosynthesis and metabolism, metabolism of terpenoids and polyketides, and biosynthesis of other secondary metabolites (Fig. 6). The dominant bacterial genera contributing to the antibiotic resistance were *Acinetobacter*, *Bacillus*, *Bacteroides*, *Bifidobacterium*, *Blautia*, *Clostridium*, *Corynebacterium*, *Erwinia*, *Flexispira*, *Halomonas*, *Klebsiella*, *Megamonas*, *Megasphaera*, *Parabacteroides*, *Proteus*, *Pseudomonas*, *Ruminococcus*, *Sporolactobacillus* and *Wautersiella*. Genes encoding for flagellin was associated with the genera *Bacillus*, *Clostridium*, *Erwinia*, *Flexispira*, *Halomonas*, *Lysinibacillus*, *Megamonas*, *Oscillospira*, *Proteus*, *Pseudomonas*, *Ruminococcus*, SMB53, *Solibacillus* and *Sporolactobacillus*. Genes encoding for Lipid A synthesis were deduced to occur primarily in *Acinetobacter*, *Bacteroides*, *Bifidobacterium*, *Corynebacterium*, *Dialister*, *Erwinia*, *Flexispira*, *Halomonas*, *Klebsiella*, *Megamonas*, *Megasphaera*, *Parabacteroides*, *Prevotella*, *Proteus*, *Pseudomonas* and *Wautersiella* (Table 3).

**Table 3:** PICRUSt predicted genes and corresponding bacterial genus involved in antibiotic resistance and pro-inflammatory activities in *sa-um*

Pro-inflammatory genes					Antibiotic resistance genes				
K00748	K02406	K02517	K11693	K11694	K05595	K07552	K08170	K07694	K08221
*Acinetobacter*	*Bacillus*	*Acinetobacter*	*Candidatus Arthromitus*	*Staphylococcus*	*Bacteroides*	*Acinetobacter*	*Clostridium*	*Ruminococcus*	*Bacillus*
*Akkermansia*	*Clostridium*	*Bacteroides*	*Staphylococcus*		*Bifidobacterium*	*Bacteroides*	*Klebsiella*	*Blautia*	*Sporolactobacillus*
*Bacteroides*	*Erwinia*	*Bifidobacterium*	*Exiguobacterium*		*Erwinia*	*Clostridium*			
*Dialister*	*Flexispira*	*Corynebacterium*			*Klebsiella*	*Corynebacterium*			
*Erwinia*	*Halomonas*	*Dialister*			*Parabacteroides*	*Erwinia*			
*Flexispira*	*Lysinibacillus*	*Erwinia*			*Proteus*	*Flexispira*			
*Halomonas*	*Megamonas*	*Flexispira*			*Pseudomonas*	*Halomonas*			
*Klebsiella*	*Oscillospira*	*Halomonas*			*Wautersiella*	*Klebsiella*			
*Megamonas*	*Proteus*	*Klebsiella*				*Megamonas*			
*Megasphaera*	*Pseudomonas*	*Megamonas*				*Megasphaera*			
*Parabacteroides*	*Ruminococcus*	*Megasphaera*				*Proteus*			
*Prevotella*	*SMB53*	*Parabacteroides*				*Pseudomonas*			
*Proteus*	*Solibacillus*	*Prevotella*				*Wautersiella*			
*Pseudomonas*	*Sporolactobacillus*	*Proteus*							
*Wautersiella*		*Pseudomonas*							
		*Wautersiella*							

### Comparison of *sa*-*um* with Italian salami

**Fig. 7:**
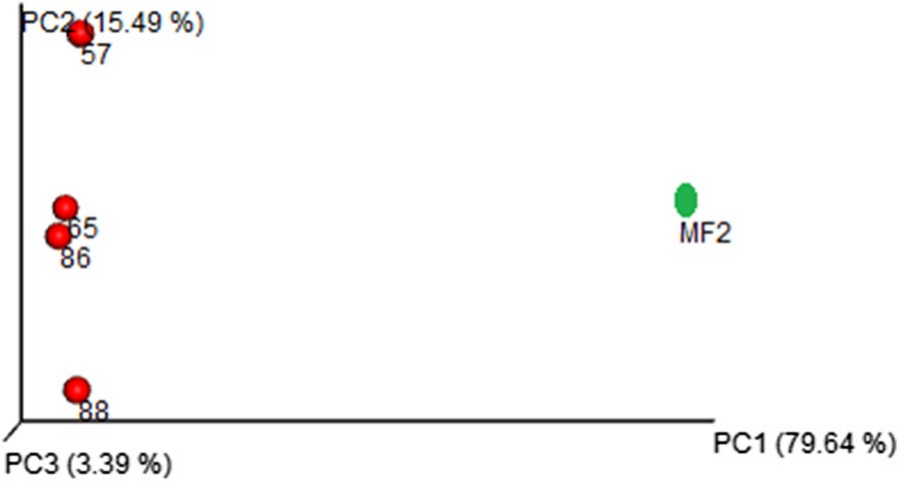
Comparison of bacterial community of *sa*-*um* and Italian salami. All the representatives of Italian salami (Red) are far apart from the *sa*-*um* sample (green) in PCoA plot generated using the unweighted Unifac distance (57, 65, 86, 88 denotes fastq file SRR5298557, SRR5298558, SRR5298588, SRR5298586, respectively)

Four fermented pork (Italian Salami) meta-genome samples were compared with *sa*-*um* microbiota in order to ascertain the bacterial community of fermented products. The resultant analysis revealed that these *sa*-*um* microbiota populations are quite different in terms of composition and the relative proportion of bacterial communities. It was found that both Italian salami and *sa*-*um* were dominated with phylum *Firmicutes* and *Proteobacteria*. However, the Italian salami was dominated by the class *Bacilli*, whereas *sa*-*um* was enriched with *Clostridia*. The beta diversity analysis using unweighted Unifac approach also revealed that the *sa*-*um* contained unique bacterial communities in comparison with the Italian salami samples (Fig. 7).

## Discussion

Microbial food safety and preservation are the important criteria for the consumers and allied industries. The food borne illnesses and hazardous intoxications by undesired microbial populations is still a matter of concern (EFSA and ECDC, 2013). The purpose of the study was to analyze the chemical composition and the bacterial diversity of fermented pork fat *sa*-*um.* Illumina sequencing was used to capture the complete bacterial diversity in order to assess the microbial safety of *sa*-*um*.

Higher fat content in *sa*-*um* is due to exclusive use of pork caul fat as the raw material. The observation of monoacyl and diacylglycerides in addition to triacylglycerides in *sa*-*um* is indicative of the enzymatic lipolytic activity by the microbial populations. It is probable that the enzymatic lipolytic activity of microbial populations could have rendered the peculiar flavour and characteristics of *sa*-*um*. Although the free fatty acids were not detected in this preliminary study, presence of free fatty acids is not ruled out as the lipids were extracted from *sa*-*um* using dicholoromethane:methanol or acetonitrile:methanol as solvent systems (Schieber 2008; Lorenzo and Franco 2012). The observed class composition of lipids in *sa*-*um* with high levels of sterols, triacylglycerols (with high levels of saturated fatty acids) and phospholipids is the characteristics of lard (Indrasti et al. 2010).

Although fermented food is a regular ingredient in the diet of many communities around the world, yet their indigenous production remains crude and rudimentary. Fermentation under largely unhygienic conditions results in the reduction in the beneficial attributes of fermentation and often leads to the growth of harmful pathogens, thereby constituting microbiological hazards. However, no inoculation of starter culture is being used for *sa*-*um* production. The anaerobic fermentation inside the closed vessel might play a role in eliminating the growth of aerobic spoilage bacteria. In the present study, majority of the classified reads belonged to the phylum Firmicutes. A total of 72 bacterial genera were identified, indicating the presence of complex and abundant bacterial communities. The metabolic by-products of these abundant bacterial members plays a key role in the formation of its unique flavours (Huang et al. 2017).

To ensure the safety and appeal of indigenous food products, it is necessary to determine presence of pathogens in them. Highest numbers of OTU falls under the genus *Clostridium*. This may be due to fact that the higher fat content, as a result of the lipolytic processes, may be creating an anaerobic environment and thereby inhibiting most of the aerobic microorganisms. Six *Clostridium* species was identified in *sa*-*um* were *C. butyricum*, *C. citroniae*, *C. methylpentosum*, *C. perfringens*, *C. saccharogumia* and *C. tetani.* In general, *C. butyricum* is used as a probiotics agent and employed to treat antimicrobial and non-antimicrobial associated diarrhea, constipation, and irritable bowel syndrome (Seki et al. 2003; Shimbo et al. 2005). However, both *C. perfringens* is a major concern in meat or fermented meat products (Akhtar et al. 2009; Golden et al. 2009; Linton et al. 2014).

The present study also identified the genus *Helicobacter* in *sa*-*um*. Some members under this genus are pathogenic and found to cause peptic ulcers, gastritis, and stomach cancer (Yamaoka 2008). This is a significant risk factor for high incidence of stomach cancer among the populace of Mizoram (Ghatak et al. 2016). Identified bacterial genera possibly involved in fermentation of *sa*-*um* were *Kouria*, *Lactobacillus*, *B. coagulans*, *Collinsella*, and *Coprococcus*. The genus *Kouria* is a usually considered as non-pathogenic bacteria which are rarely associated with human infections (Nam et al. 2012). *B. coagulans* is a lactic acid-forming species having the property of both genera *Lactobacillus* and *Bacillus* and is often used as probiotics in pigs, cattles and shrimp (Hammer 1915; Sanders et al. 2003). A member under the genus *Collinsella* was capable of fermenting carbohydrates and thus produces hydrogen gas and ethanol (Goldman 2015). The anaerobic cocci, *Coprococcus* actively ferments carbohydrates and produces butyric, acetic, formic and/or lactic acids. Veillonella are Gram-negative anaerobic cocci, well known for its lactate fermenting abilities. However, the observed mild acidic pH of *sa*-*um* (pH = 6.60) is indicative of the production of organic acids at modest levels due to the fermentation of negligible carbohydrate content in raw material and/or *sa*-*um*. Recently, the informative software PICRUSt was used to study the functional potential of the bacterial community using 16S rRNA gene information (Langille et al. 2013). The imputed metagenomic analysis of the *sa*-*um* metagenome identifies antibiotic resistance genes and pro-inflammatory molecules mainly arising from the bacterial families such as *Bacillaceae*, *Bacteroidaceae*, *Clostridiaceae*, *Corynebacteriaceae* and *Enterobacteriaceae* indicative of the presence of human pathogenic species in *sa*-*um* (Tyx et al. 2016). Resistant gene might have occurred within the gut microbiota of pig and thus serve as a potential route of antimicrobial resistance transmission from animal to human microbiota (Fraqueza 2015). Presence of these resistant genes could be a major concern for this geographic region.

The introduction of NGS technology significantly improved the study of bacterial community present in autochthonous fermented food and the metabolic activity of these microbiota is the key criterion for its unique characteristic sensory attributes. Earlier studies on Italian salami identified the presence of 32 different *Staphylococcus* and 33 *Lactobacillus* species from different producers (Połka et al. 2014). Comparison analysis for *sa*-*um* with Italian salami revealed that most of the bacterial orders in Italian salami belonged to *Lactobacillus*, whereas in *sa*-*um* it was only 0.3%. This difference may be due to the variation in the carbohydrate or fat content of raw materials used for fermentation in *sa*-*um* and Italian salami. This is the first report of characterization of *sa*-*um* a traditional fermented pork fat food. In the present study, high fat content and large number pathogens in *sa*-*um* were observed which may cause adverse health risks. This scientific work on an autochthonous fermented pork fat will be useful to the consumers by providing essential and important information on the benefits as well as potential health risks associated with the traditional foodstuff.

## Additional file

**Additional file 1: Figure S1.** HP-TLC (CAMAG Linomat 5) Chromatogram of *sa*-*um* extract (10 µl) with Scanning wavelength of 280 nm. **Figure S2.** Distribution of microbial composition between *sa*-*um* and Italian Salami. Studies compared at A: Phylum level and B: Order level.

## Abbreviation

ESI^**+**^-MS: electrospray ionization-mass spectrometry
