# Supplementary material for: Metagenomic analysis and the functional profiles of traditional fermented pork fat ‘sa-um’ of Northeast India
Source: AMB Express. 2018 Oct 8;8:163. doi: 10.1186/s13568-018-0695-z (PMC6175732; doi:10.1186/s13568-018-0695-z)
Supplement: Supplementary file 1 — Additional file 1: Figure S1. HP-TLC (CAMAG Linomat 5) Chromatogram of sa-um extract (10 µl) with Scanning wavelength of 280 nm. Figure S2. Distribution of microbial composition between sa-um and Italian Salami. Studies compared at A: Phylum level and B: Order level. [file 13568_2018_695_MOESM1_ESM.doc]

**Supplementary Figures**

Figure S1. HP-TLC (CAMAG Linomat 5) Chromatogram of Sa-um extract (10 µl) with Scanning wavelength of 280 nm


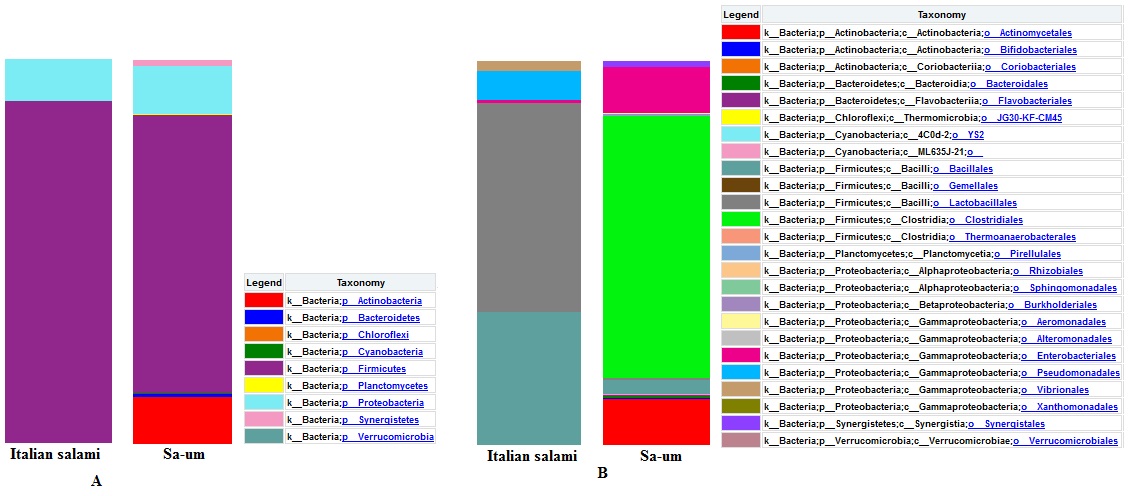


**Figure S2. Distribution of microbial composition between *sa-um* and Italian Salami.** Studies compared at A: Phylum level and B: Order level
